# Involvement of abscisic acid-responsive element-binding factors in cassava (*Manihot esculenta*) dehydration stress response

**DOI:** 10.1038/s41598-019-49083-3

**Published:** 2019-09-02

**Authors:** Ren-Jun Feng, Meng-Yun Ren, Li-Fang Lu, Ming Peng, Xiao Guan, Deng-Bo Zhou, Miao-Yi Zhang, Deng-Feng Qi, Kai Li, Wen Tang, Tian-Yan Yun, Yu-Feng Chen, Fei Wang, Dun Zhang, Qi Shen, Ping Liang, Yin-Dong Zhang, Jiang-Hui Xie

**Affiliations:** 1 Key Laboratory of Biology and Genetic Resources of Tropical Crops, Ministry of Agriculture, Institute of Tropical Bioscience and Biotechnology, Chinese Academy of Tropical Agricultural Sciences (CATAS), Haikou, 571101 P.R. China; 2 Institute of Crops and Nuclear Technology Utilization, Zhejiang Academy of Agricultural Sciences, Hangzhou, 310021 P.R. China; 3 Department of Medical Physiology, Hainan Medical University, Haikou, 571199 P.R. China; 4 College of Agronomy, Hainan University, Haikou, 570228 P.R. China; 5 Chinese Research Academy of Environmental Sciences, Beijing, 100012 P.R. China

**Keywords:** Abiotic, Drought

## Abstract

Cassava (*Manihot esculenta*) is a major staple food, animal feed and energy crop in the tropics and subtropics. It is one of the most drought-tolerant crops, however, the mechanisms of cassava drought tolerance remain unclear. Abscisic acid (ABA)-responsive element (ABRE)-binding factors (ABFs) are transcription factors that regulate expression of target genes involved in plant tolerance to drought, high salinity, and osmotic stress by binding ABRE *cis*-elements in the promoter regions of these genes. However, there is little information about *ABF* genes in cassava. A comprehensive analysis of *Manihot esculenta ABFs* (*MeABFs*) described the phylogeny, genome location, *cis*-acting elements, expression profiles, and regulatory relationship between these factors and *Manihot esculenta betaine aldehyde dehydrogenase genes* (*MeBADHs*). Here we conducted genome-wide searches and subsequent molecular cloning to identify seven *MeABFs* that are distributed unevenly across six chromosomes in cassava. These *MeABFs* can be clustered into three groups according to their phylogenetic relationships to their *Arabidopsis* (*Arabidopsis thaliana*) counterparts. Analysis of the 5′-upstream region of *MeABFs* revealed putative *cis*-acting elements related to hormone signaling, stress, light, and circadian clock. *MeABF* expression profiles displayed clear differences among leaf, stem, root, and tuberous root tissues under non-stress and drought, osmotic, or salt stress conditions. Drought stress in cassava leaves and roots, osmotic stress in tuberous roots, and salt stress in stems induced expression of the highest number of *MeABFs* showing significantly elevated expression. The glycine betaine (GB) content of cassava leaves also was elevated after drought, osmotic, or salt stress treatments. BADH1 is involved in GB synthesis. We show that *MeBADH1* promoter sequences contained ABREs and that *MeBADH1* expression correlated with *MeABF* expression profiles in cassava leaves after the three stress treatments. Taken together, these results suggest that in response to various dehydration stresses, MeABFs in cassava may activate transcriptional expression of *MeBADH1* by binding the *MeBADH1* promoter that in turn promotes GB biosynthesis and accumulation via an increase in *MeBADH1* gene expression levels and MeBADH1 enzymatic activity. These responses protect cells against dehydration stresses by preserving an osmotic balance that enhances cassava tolerance to dehydration stresses.

## Introduction

Cassava (*Manihot esculenta*) is a perennial euphorbiaceous shrub grown mainly for its starchy tubers, which are used as food, animal feed, and in non-food products^1–3^. With its inherent high drought and poor nutrients tolerance, as well as high starch content, cassava is a critical resource^2–7^. However, the mechanisms underlying the stress tolerance of cassava remain unclear. Drought is a major abiotic stress that can reduce crop output^8^. In some areas that experience unpredictable water shortages, enhancement of plant resistance or tolerance to drought is important for minimizing crop losses^8^. Various strategies to combat drought stress include maintaining water status through rapid stomatal closure and changes in growth and development patterns^9^. Both rapid stomatal movement and integrated growth plasticity involve long distance communication between different organs that is primarily mediated by the stress-related phytohormone abscisic acid (ABA)^9^.

ABA is indispensable for plant growth and development^10^. ABA regulates a variety of plant developmental processes such as leaf senescence, seed maturation and dormancy, bud dormancy, and adaptive responses to abiotic and biotic stresses, in particular drought and salinity, by controlling stomata closure, osmotic potential, and/or wax deposition^11,12^. In one verified model for ABA signaling, the presence of ABA is sensed by the pyrabactin resistance (PYR)/PYR-like (PYL)/regulatory components of the ABA receptor (RCAR)^13–15^. ABA ligation to the RCAR receptor induces a conformational change in members of the PYR/PYL/RCAR receptor family, rendering them able to bind and inhibit type 2C protein phosphatases (PP2C) and in turn release inhibition of sucrose non-fermenting 1-related protein kinase 2 (SnRK2)^13–15^. ABA-activated SnRK2 then phosphorylates ABA-responsive element (ABRE)-binding factors (ABFs) such as ABF2 to induce upregulation of ABA-responsive gene expression^13–15^.

ABF is a family of ABA-dependent bZIP transcription factors that interact with the ABRE (PyACGTGG/TC), a conserved *cis*-acting regulatory sequence found in the 5′ flanking regions of many ABA-responsive genes in plants^16–18^. The *ABF* family genes *ABF1*, *ABF2*, *ABF3*, and *ABF4* are mainly expressed in vegetative tissues of *Arabidopsis* exposed to abiotic stress conditions^19^. Whereas *ABF1* expression is significantly induced by cold but not osmotic stress^20^, expression of the other three *ABF* genes (*ABF2*, *ABF3*, and *ABF4*) is induced both by ABA and dehydration stresses such as drought and high salinity. These three factors have been shown to be key regulators of ABA signaling in response to osmotic stress^20^, as was found in *Gossypium hirsutum* in which overexpression of exogenous and endogenous *ABFs* substantially increase drought tolerance, primarily through stomatal regulation that reduces transpiration and photosynthetic productivity^21^.

Although *ABFs* play a significant role in responses to abiotic stresses in plants, little is known about the exact role *ABFs* play in cassava. To explore the role of *Manihot esculenta ABFs* (*MeABFs*) in drought tolerance of cassava, we identified and characterized candidate *MeABFs*. Using bioinformatic methods, these genes were localized in the cassava genome, constructed as phylogenetic trees, and analyzed for stress-responsive *cis*-elements in the promoter regions. We then investigated the expressional patterns of *MeABFs* in different cassava tissues after exposure to drought, PEG, or high salt. Drought, osmotic, or salt stresses in one or two cassava tissues induced significantly elevated expression of *MeABFs*.

Glycine betaine (GB), a bipolar quaternary ammonium compound that acts as an osmoprotectant, accumulates in many plant species in response to various abiotic stresses^22^. GB protects plants from abiotic stresses by maintaining the osmotic balance under various abiotic stresses and by stabilizing the quaternary structure of complex proteins^23^. We observed increases in GB content in cassava following exposure to drought, osmotic, or salt stress treatments. Betaine aldehyde dehydrogenase (BADH) converts betaine aldehyde into GB in plants^24^. As such, we speculated that *MeABF* and *Manihot esculenta BADH* (*MeBADH*) levels might be correlated. We further found that the promoter regions of *MeBADH1* contain ABRE cis-acting element that could interact with ABFs. Indeed, *MeBADH* expression profiles in cassava exposed to drought, osmotic, or salt stress tended to be similar to those for *MeABF* genes. These results suggest that *MeABFs* may affect GB content by regulating *MeBADH* gene expression that in turn confers drought tolerance to cassava. In this study, we provide a theoretical foundation for further explanation of molecular mechanisms involved in cassava drought tolerance, and provide a basis for cultivation of novel drought-tolerance cassava varieties and breeding resources.

## Results

### *MeABF* identification and phylogenetic analysis

Seven members of the *MeABF* family were identified in the cassava cultivar South China 5 (SC5) by BLAST searches of the Phyzotome database and NCBI database. The resulting genes, termed *MeABF1*, *MeABF2*, *MeABF3*, *MeABF4*, *MeABF5*, *MeABF6*, and *MeABF7*, were then cloned and sequenced, and their sequences were aligned with the counterparts of the cassava cultivar AM560-2.

To examine phylogenetic relationships among ABFs, a phylogenetic tree was constructed using the amino acid sequences of ABFs from *Arabidopsis* and cassava (Supplementary Fig. S1 and Table S1). The phylogenetic tree revealed that the seven MeABFs could be classified into three groups (A, B, and C). Group A MeABFs underwent a significant expansion, as two MeABFs (MeABF2 and MeABF3) existed in cassava, whereas one corresponding *Arabidopsis* ortholog (*Arabidopsis thaliana* ABF2, AtABF2) were present. The size of Group B MeABFs decreased as evidenced by a single MeABF1 and three AtABFs (AtABF1, AtABF3, and AtABF4). Surprisingly, Group C included four MeABFs (MeABF4, MeABF5, MeABF6, and MeABF7) that were exclusive to cassava. Furthermore, MeABF2 and MeABF3, and MeABF5 and MeABF6 showed high sequence similarity with each of the other among seven MeABFs.

### *MeABF* chromosomal distribution and *cis*-acting element analysis

**Figure 1:**
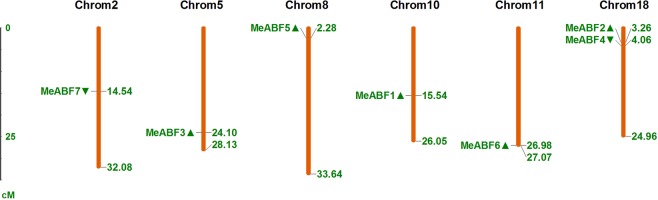
Distribution of *MeABFs* on cassava chromosomes. The chromosome numbers and size are indicated at the top and bottom of each bar, respectively. The numbers on the right side of the bars designated the approximate physical positions of the first exon of corresponding *MeABFs* on cassava genome. The triangles next to gene names show the transcription directions.

To determine the chromosomal distribution of the identified *MeABFs*, we subjected the genomic sequences of the seven *MeABFs* to BLAST analysis against the cassava genome database. The location image shows that the seven *MeABFs* could be successfully mapped to six of the 18 chromosomes present in the cassava genome (Fig. 1). *MeABF2* and *MeABF4* were distributed on chromosome 18, and *MeABF1*, *MeABF3*, *MeABF5*, *MeABF6*, and *MeABF7* were present on chromosomes 10, 5, 8, 11, and 2, respectively.

To further explore transcriptional regulation of *MeABFs*, the promoter sequence *cis*-elements of these genes were predicted using PlantCare software (Supplementary Table S2). Previous studies showed that promoters for most *MeABFs* contained one or more *cis*-elements of genes that act in hormone signaling pathways involving ABA (ABRE), jasmonic acid (CGTCA-motif and TGACG-motif), gibberellic acid (GARE-motif, P-box, and TATC-box), salicylic acid (TCA-element), and auxin (TGA-element). In this study, we found that the upstream regions of most of the identified *MeABFs* contained one or more *cis*-elements related to stress responses. These elements include Box-W1 (fungal elicitor responsive element), HSE (heat stress response element), LTR (low-temperature response element), MBS (MYB binding site involved in drought-inducibility), TC-rich repeats (*cis*-acting element involved in defense and stress responsiveness), W box (WRKY binding site involved in abiotic stress responsiveness), and box S (elicitor-responsive element involved in the wounding and pathogen responsiveness). Interestingly, the promoter regions of most *MeABFs* contain one element involved in circadian clock and additional elements involved in light responsiveness. The *cis*-regulatory elements present in the 5′ upstream regions of the *MeABFs* identified here suggested that they play an important mediating role in plant growth and development, as well as in responses to various stresses.

### *MeABF* expression levels in cassava leaves under dehydration stresses

**Figure 2:**
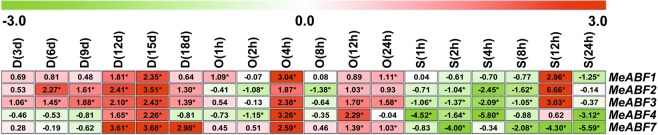
Expression profiles of MeABFs in cassava leaves under three stress conditions. Relative expression values for the target genes were calculated according to the 2^−ΔΔCt^ method. The heatmap was generated based on the log_2_ of relative expression values using MeV software. Red, green and white indicate up-regulated, down-regulated and unchanged expression, respectively. Asterisk on the right corner of the value indicates a statistically significant difference at *P* -value < 0.05 and the absolute value of log_2_ relative expression values >1. D, drought stress; O, osmotic stress; S, salt stress.

**Figure 3:**
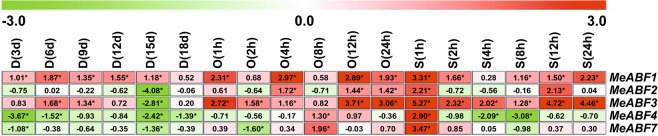
Expression profiles of MeABFs in cassava stems under three stress conditions. Relative expression values for the target genes were calculated according to the 2^−ΔΔCt^ method. The heatmap was generated based on the log_2_ of relative expression values using MeV software. Red, green and white indicate up-regulated, down-regulated and unchanged expression, respectively. Asterisk on the right corner of the value indicates a statistically significant difference at *P* -value < 0.05 and the absolute value of log_2_ relative expression values >1. D, drought stress; O, osmotic stress; S, salt stress.

**Figure 4:**
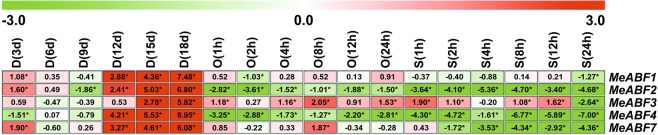
Expression profiles of MeABFs in cassava roots under three stress conditions. Relative expression values for the target genes were calculated according to the 2^−ΔΔCt^ method. The heatmap was generated based on the log_2_ of relative expression values using MeV software. Red, green and white indicate up-regulated, down-regulated and unchanged expression, respectively. Asterisk on the right corner of the value indicates a statistically significant difference at *P* -value < 0.05 and the absolute value of log_2_ relative expression values >1. D, drought stress; O, osmotic stress; S, salt stress.

**Figure 5:**
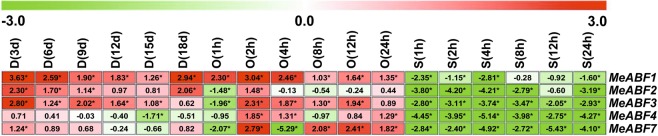
Expression profiles of MeABFs in cassava tuberous roots under three stress conditions. Relative expression values for the target genes were calculated according to the 2^−ΔΔCt^ method. The heatmap was generated based on the log_2_ of relative expression values using MeV software. Red, green and white indicate up-regulated, down-regulated and unchanged expression, respectively. Asterisk on the right corner of the value indicates a statistically significant difference at *P* -value < 0.05 and the absolute value of log_2_ relative expression values > 1. D, drought stress; O, osmotic stress; S, salt stress.

All of the measured *MeABF* genes of cassava leaf tissue showed differential expression profiles after drought, osmotic, or salt stress treatments (Fig. 2), except for *MeABF5* and *MeABF6*, whose expression levels were too low to detect. This phenomenon also occurred in stems, roots and tuberous roots (Figs 3–5).

The transcript levels of the *MeABF1*, *MeABF2*, *MeABF3*, *MeABF4*, and *MeABF7* of cassava leaves were significantly elevated or remained at a similar level as the control at all points in time in response to drought stress (Fig. 2, Supplementary Tables S3 and S4), with significant up-regulation by 33.33%, 83.33%, 100.00%, 33.33%, and 50.00%, respectively (Supplementary Table S5).

Compared to the control, the expression of *MeABF1*, *MeABF2*, *MeABF3*, *MeABF4*, and *MeABF7* was significantly increased or unchanged in contrast to the control at any point in time in cassava leaves treated by osmotic stress, with the exception of *MeABF2* (2 h and 8 h) and *MeABF4* (2 h), which were suppressed at one or two points in time (Fig. 2, Supplementary Tables S3 and S4). The observed significant increases relative to control were 50.00%, 33.33%, 50.00%, 33.33%, and 50.00% for *MeABF1*, *MeABF2*, *MeABF3*, *MeABF4*, and *MeABF7*, respectively (Supplementary Table S5).

The expression levels of *MeABF1*, *MeABF2*, and *MeABF3* in cassava leaves were significantly increased only at 12 h, whereas the levels for *MeABF1*, *MeABF2*, *MeABF3*, *MeABF4*, and *MeABF7* were significantly reduced or sustained at a similar level as the control at most time points by salt stress (Fig. 2, Supplementary Tables S3 and S4). The percentage change relative to control was 16.67%, 16.67%, 16.67%, 0%, and 0% for *MeABF1*, *MeABF2*, *MeABF3*, *MeABF4*, and *MeABF7*, respectively, significantly induced by salt stress (Supplementary Table S5).

In cassava leaves, drought stress significantly elevated the expression of the highest number of *MeABFs* (60.00%), followed by osmotic stress (43.33%) and then salt stress (10.00%, Supplementary Table S5).

### *MeABF* expression levels in cassava stems under dehydration stresses

In cassava stems, *MeABF1* and *MeABF3* expression levels were significantly up-regulated or sustained at a level similar to that of the control throughout the drought treatment period, except at 15 d when *MeABF3* levels were significantly reduced. *MeABF2*, *MeABF4*, and *MeABF7* expression was significantly decreased or unchanged at any point in time under drought stress (Fig. 3, Supplementary Tables S6 and S7). In addition, *MeABF1* and *MeABF3* expression was significantly upregulated by 83.33% and 33.33%, respectively, relative to control (Supplementary Table S8).

After osmotic stress treatment, *MeABF1*, *MeABF2*, *MeABF3*, *MeABF4*, and *MeABF7* expression in cassava stems was significantly increased or unchanged relative to the control at all points in time, with the exception of *MeABF7* levels at 2 h, which were significantly reduced (Fig. 3, Supplementary Tables S6 and S7). Expression of these five *MeABFs* was significantly induced relative to control by 66.67%, 50.00%, 83.33%, 16.67%, and 16.67%, respectively (Supplementary Table S8).

For salt stress treatment, *MeABF1*, *MeABF2*, *MeABF3*, and *MeABF7* expression in cassava stems was significantly elevated or remained at a similar level as the control, whereas *MeABF4* expression was significantly suppressed at any point in time except for at 1 h, when expression was significantly up-regulated (Fig. 3, Supplementary Tables S6 and S7). *MeABF1*, *MeABF2*, *MeABF3*, *MeABF4*, and *MeABF7* expression was significantly elevated by 83.33%, 33.33%, 100.00%, 16.67%, and 16.67%, respectively, relative to the control (Supplementary Table S8).

Salt stress significantly elevated the expression of the highest number of *MeABFs* (50.00%) in cassava stems, followed by osmotic stress (46.67%) and then drought stress (23.33%, Supplementary Table S8).

### *MeABF* expression levels in cassava roots under dehydration stresses

In cassava roots, transcript levels of *MeABF1*, *MeABF2*, *MeABF3*, *MeABF4*, *and MeABF7* were significantly increased or sustained at a level similar to that of the control by drought stress, with the exception of *MeABF2* and *MeABF4*, which were significantly reduced at only one point in time (9 d and 1 d, respectively; Fig. 4 and Supplementary Tables S9 and S10). The expression of these five was significantly induced by 66.67%, 66.67%, 33.33%, 50.00%, and 66.67%, respectively, relative to the control (Supplementary Table S11).

*MeABF3* and *MeABF7* expression in cassava roots was significantly up-regulated or unchanged in contrast to the control in response to osmotic stress. The expression levels of *MeABF1*, *MeABF2*, *and MeABF4* were significantly decreased or sustained at a level similar to that of the control at any point in time under osmotic stress (Fig. 4, Supplementary Tables S9 and S10). The rate of significant up-regulation for *MeABF3* and *MeABF7* was 66.67% and 16.67%, respectively (Supplementary Table S11).

Under salt stress, *MeABF3* expression was significantly induced (66.67%) or remained at a level similar to that of the control in cassava roots, except at 24 h, when the level was significantly suppressed. The expression levels of *MeABF1*, *MeABF2*, *MeABF4*, and *MeABF7* in cassava roots were significantly reduced or unchanged at any point in time by salt stress (Fig. 4, Supplementary Tables S9–11).

In cassava roots, drought stress significantly elevated the expression of the highest number of *MeABFs* (56.67%), followed by osmotic stress (16.67%) and then salt stress (13.33%, Supplementary Table S11).

### *MeABF* expression levels in tuberous roots under dehydration stresses

The transcript levels of *MeABF1*, *MeABF2*, *MeABF3*, and *MeABF7* in cassava tuberous roots were significantly increased or sustained at a level similar to that of the control at all points in time by drought stress. *MeABF4* expression levels in tuberous roots were significantly reduced or unchanged after drought stress treatment (Fig. 5, Supplementary Tables S12 and S13). The induction rate for *MeABF1*, *MeABF2*, *MeABF3*, and *MeABF7* was 100.00%, 66.67%, 83.33% and 16.67%, respectively (Supplementary Table S14).

*MeABF1*, *MeABF2*, *MeABF3*, *MeABF4*, and *MeABF7* expression in cassava tuberous roots was significantly up-regulated or unchanged relative to the control at any point in time by osmotic stress treatment, with the exception of *MeABF2* (1 h), *MeABF3* (1 h), and *MeABF7* (1 h and 4 h), which were significantly down-regulated at one or two points in time (Fig. 5, Supplementary Tables S12 and S13). The rate of significant induction of expression was 100.00%, 16.67%, 66.67%, 50.00%, and 66.67%, respectively (Supplementary Table S14).

After salt stress treatment, *MeABF1*, *MeABF2*, *MeABF3*, *MeABF4*, and *MeABF7* expression levels in tuberous roots were significantly reduced at any point in time (Fig. 5, Supplementary Tables S12 and S13).

Osmotic stress significantly elevated the expression of the highest number of *MeABFs* (60.00%) in cassava tuberous roots, followed by drought stress (53.33%) and then salt stress (0.00%; Supplementary Table S14).

### Measurement of GB content in cassava

**Figure 6:**
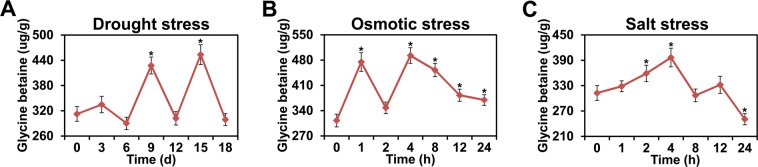
GB content s in cassava fresh leaves after drought (**A**), osmotic (**B**), or salt (**C**) stress treatments. Y-axes indicate GB concentration and error bars denote standard deviation (*P* -value < 0.05).

We also measured GB content in the fresh leaves of cassava that had been treated by drought, osmotic, or salt stress (Fig. 6, Supplementary Tables S15 and S16). The GB content was significantly altered by the three stresses and similar patterns manifested as an initial increase, a decrease, and then an increase and a decrease. Furthermore, GB levels increased and then decreased during the final stages under drought stress conditions. GB exhibited a maximum accumulation at 4 h after osmotic and salt stress treatments and at 15 d after drought stress treatment. Overall, GB content was elevated under drought, osmotic, and salt stress conditions in cassava leaves (Supplementary Table S17).

### Identification of *MeBADH*s, *cis*-acting elements, and expression analysis

**Figure 7:**
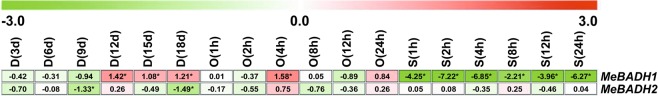
Expression profiles of MeBADHs in cassava leaves under three stress conditions. Relative expression values for the target genes were calculated according to the 2^−ΔΔCt^ method. The heatmap was generated based on the log_2_ of relative expression values using MeV software. Red, green and white indicate up-regulated, down-regulated and unchanged expression, respectively. Asterisk on the right corner of the value indicates a statistically significant difference at *P* -value < 0.05 and the absolute value of log_2_ relative expression values > 1. D, drought stress; O, osmotic stress; S, salt stress.

Two members of the *MeBADH* family, termed *MeBADH1* and *MeBADH2*, were identified in cassava using the same methods as those used to identify *MeABFs*. *Cis*-acting element analysis in the promoter sequence of these two genes showed that the upstream region of *MeBADH1* contained three types of *cis*-elements that act in hormone signaling pathways involving ABA (ABRE), gibberellin (GARE-motif), and auxin (TGA-element), whereas the *MeBADH2* promoter contained only the ERE cis-element (ethylene-responsive element) (Supplementary Table S18). The *MeBADH1* and *MeBADH2* promoter regions both contained two or more kinds of *cis*-elements related to stress responses, and seven or more kinds of *cis*-elements involved in light responsiveness. The *MeBADH1* promoter also contained a circadian *cis*-element. *MeBADH1* and *MeBADH2* showed differential expression patterns in cassava leaf tissue after drought, osmotic, and salt stress treatments (Fig. 7; Supplementary Tables S19 and S20). *MeBADH1* expression was significantly induced by 50%, 16.67%, and 0% under drought, osmotic, and salt stresses, respectively. Meanwhile, induction of *MeBADH2* expression was minimal in response to all three stresses (Supplementary Table S21).

## Discussion

### Effect of dehydration stresses on *MeABF* transcription levels in cassava

Abiotic stresses, such as drought or salinity, cause intensive losses to agricultural production worldwide^25^. Plants have evolved complex molecular, cellular, and physiological mechanisms to respond to these stresses in order to survive adverse conditions^21^. These mechanisms include signaling pathways, such as those activated by ABA, and other stress-responsive gene families^21^. ABA acts as a growth regulator in response or tolerance to abiotic stressors such as drought, salinity, cold, and heat^26–29^. The ABF family of bZIP transcription factors functions in ABA signaling pathways and plays an important role in plant responses to stresses^18,21^. Here we found that in various cassava organs, *MeABFs* exhibited differential expression patterns after drought, osmotic, and salt stress treatments(Figs 2–5), which was consistent with trends we observed in cassava in our previous study that identified genes involved in ethylene signaling in response to dehydration stresses^8^. Our results of this study showed that drought stress in cassava leaves and roots, osmotic stress in cassava tuberous roots, and salt stress in cassava stems induced expression of the highest number of *MeABFs* in terms of significantly elevated expression (Supplementary Tables S5, S8, S11 and S14). Although many of the *MeABFs* overlap with AtABF homologs in *Arabidopsis* in that their expression is induced by similar abiotic stresses and their target genes overlap, differences in their temporal and spatial expression patterns indicate that each has a unique function^16,19,30–33^.

The main negative effect of drought on plants is a shortage of water available to tissues. To reduce the amount of water lost to transpiration under drought stress, in cassava plants, leaf stomata partially close and leaf area is reduced via ABA signaling pathway, which restricts the formation of new leaves, results in leaves having a smaller size and that droop, and promotes leaf loss^34–36^. Leaf formation is regarded as an important indicator used to assess the drought tolerance of various cassava varieties^37^.

NaCl can negatively affect the energetic, hydric, and nutritional equilibria of plants^38^. Plants grown under salt stress are first affected by water stress and then by sodium and chloride ion-mediated toxicity and nutritive stresses^39^. Salinity can reduce the biomass, leaf area, and photosynthetic rate of cassava plants^40^. Therefore, NaCl not only causes the dehydration of cassava plants, similar to drought stress, but also disturbs other physiological processes and metabolic pathways related to ion homeostasis.

Unlike NaCl, which readily enters the cells to cause toxicity, PEG, a nonabsorbable, non-metabolizable and non-toxic osmotic agent, is often used because of its high molecular weight, which precludes its entry into plant cells via the cell wall^41,42^. Our results indicated that PEG affect plants through a different mechanism than that involved drought and salt stresses; this mechanism awaits further elucidation.

### Other environmental factors that affect *MeABF* transcription in cassava

Under dehydration stress, circadian clock and light conditions also appear to regulate ABA- mediated gene expression, likely conferring versatile tolerance and repressing growth under stress conditions^20^. Analysis of *cis*-acting elements in *MeABF* promoter sequences showed that *MeABF* transcription can be regulated by circadian clock, light, and hormones, except for by biotic and abiotic stresses (Supplementary Table S2).

Circadian clock is an endogenous and cell-autonomous biological timekeeper that generates roughly 24 h rhythms and provides an adaptive advantage by synchronizing physiological and metabolic processes of the plant to the external environment^43^. Plants are usually exposed to drought stress during the day, and water deficit crises would be at maximum levels near the end of the day^44^. Consequently, ABA-dependent drought responses are usually gated primarily around dusk^45^. The rhythmic expression of drought-responsive genes confers rhythmic modulation of drought responses throughout the day, as seen in *Arabidopsis* and poplar^46,47^. The ABA-inducible MYB96 transcription factor activates *TIMING OF CAB EXPRESSION 1* (*TOC1*) expression by binding directly to its gene promoter^48^. This regulation may be direct, as PSEUDO-RESPONSE REGULATOR 7 directly binds to the promoter region of *ABA DEFICIENT 1*, which encodes a zeaxanthin epoxidase involved in ABA biosynthesis^49^. The pervasive TOC1 also interacts with ABA INSENSITIVE 3 (ABI3), which has roles in ABA signaling and drought tolerance^50^. Bidirectional interactions between circadian oscillator and output pathways have also been observed in ABA-related physiological processes^51^.

ELONGATED HYPOCOTYL 5 (HY5), a constitutively-nuclear bZIP protein, was the first transcription factor that was found to promote photomorphogenesis and has been extensively studied^52,53^. HY5 physically interacts with B-box 21 (BBX21), BBX22, BBX24 and BBX25, and the bZIP domain of HY5 and the B-box domains of these BBX proteins mediate their interactions^54–59^. HY5 also promotes *BBX22* expression by directly binding to its promoter^54^, whereas BBX24 and BBX25 repress *BBX22* expression by interfering with HY5 transcriptional activity^58^. BBX21 negatively regulates *ABI5* expression by interfering with HY5 binding to the *ABI5* promoter^60^. In addition, ABI5 can directly activate its own expression, whereas BBX21 negatively regulates this activity through interactions with ABI5. These results indicate that BBX21 coordinates with HY5 and ABI5 on the *ABI5* promoter and that these transcriptional regulators work in concert to integrate light and ABA signaling in *Arabidopsis*^60^. The influence of ABA on *phototropin* expression is negligible at the mRNA level, but prominent at the protein level, and ABA appears to enhance plant sensitivity to light and promote the chloroplast avoidance response^61^. An influence of ABA on light-mediated chloroplast positioning has also been implicated in succulent plants exposed to water-deficit stress^62^.

Collectively, these observations may support the view that the circadian clock and light mediate the ability of a plant to adapt to daily changes in water status by controlling endogenous MeABF levels and subsequently *MeABF* gene expression. Interestingly, the cis-acting element ABRE is present in *MeABF* promoter sequences (Supplementary Table S2). Therefore, MeABFs could likely bind their own promoter to activate their expression as was demonstrated in studies on ABI5^60^. Furthermore, expression of two genes encoding 9-cis-epoxycarotenoid dioxygenase, a key enzyme in ABA biosynthesis, was up-regulated in response to water deficits^63^ and might be responsive to ABA itself^64^. These findings indicated that a positive feedback loop controls ABA biosynthesis and ABA signaling during water deficit stress. A more recent study found that ABFs played a role in the negative feedback regulation of ABA signaling by mediating rapid ABA-mediated induction of group A *PP2C* gene expression^65^.

### Effect of dehydration stresses on GB content in cassava leaves

To cope with harsh habitats such as high salt, drought, heat, and cold, plants have evolved various types of tolerance mechanisms, among which the accumulation of compatible solutes plays a key role in balancing the intracellular osmotic potential of plants^24^. Under conditions of water deficit or salinity stress, plant ABA levels increase dramatically, restricting water loss by stimulating stomatal closure and protecting cellular machinery against dehydration damage by promoting the accumulation of osmo-compatible solutes^44^. GB is regarded as one of the most effective compatible solutes^66^. GB can protect cells from stresses by maintaining an osmotic balance with the surrounding environment and by stabilizing the quaternary structures of complex proteins, such as antioxidant enzymes and the oxygen-evolving PSII complex^67^.

Taken together, we found that cassava leaves had increased GB concentrations in response to drought, osmotic, and salt stresses (Fig. 6, Supplementary Table S17). Furthermore, osmotic stress had the largest impact on the levels of GB content followed by drought stress and then salt stress (Supplementary Table S17). These results suggest that cassava plants may induce expression of *BADH* genes via the ABA signaling pathway to increase the GB content and adapt to harsh habitats.

### MeABF regulation of *MeBADH* transcription

In higher plants, BADH catalyzes the key step of GB biosynthesis^23^. In *Ammopiptanthus nanus*, endogenous expression of *AnBADH* is strongly induced by exposure to high salt, drought, ABA, heat, or cold^24^. Heterologous expression of *AnBADH* in *Arabidopsis* enhanced its tolerance to high salt and drought stresses, suggesting that BADH could play a critical role in plant abiotic tolerance through ABA signaling pathways^24^. An incremental increase in BADH activity induced by ABA treatment of maize also promoted increases in GB content under drought stress^68^. Activated SnRK2s phosphorylate and activate BADH, as do scavenging reactive oxygen species and many other proteins related to abiotic tolerance^69–71^. Therefore, we speculate that ABFs may activate BADH production in plants after ABFs are phosphorylated and activated by SnRK2s via the ABA signaling pathway.

**Figure 8:**
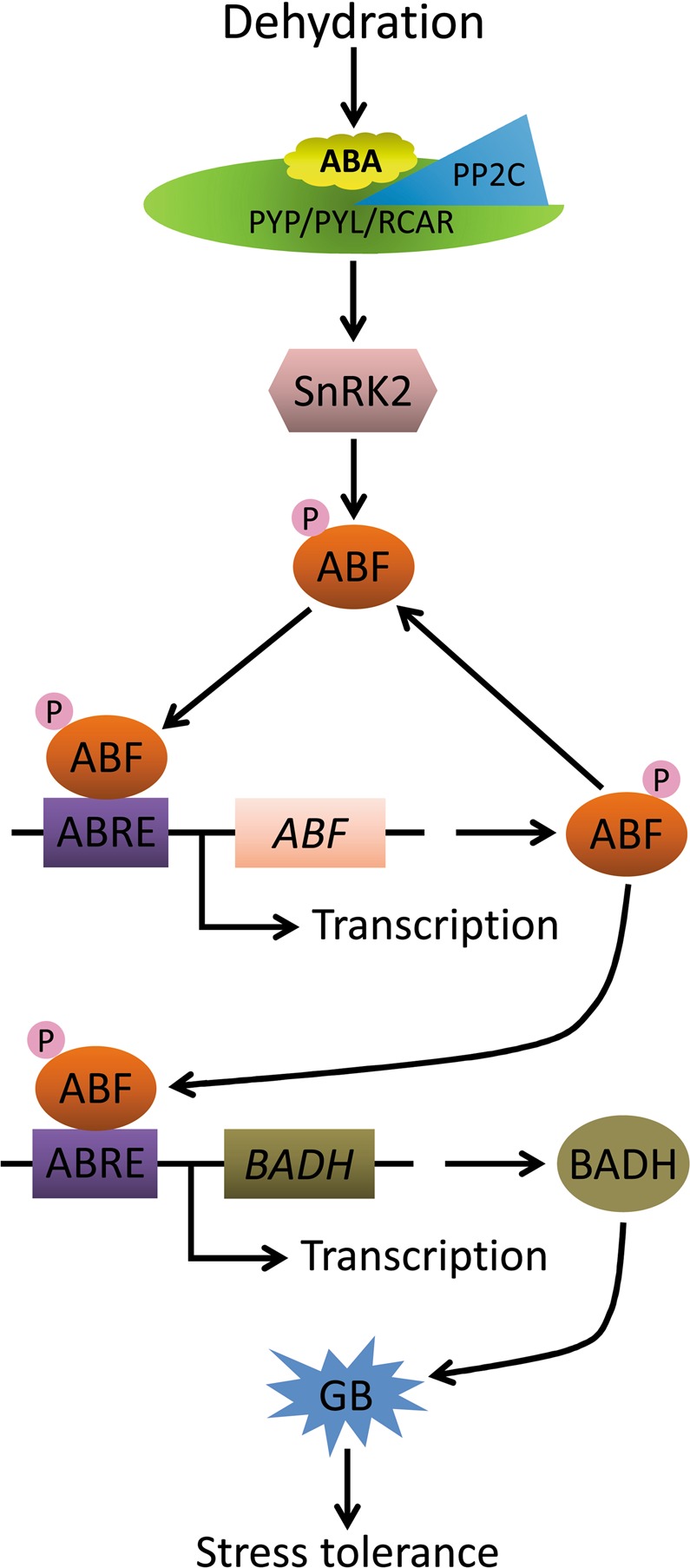
Proposed mechanism by which ABF promotes plant tolerance to dehydration stress^65^. In response to dehydration stress, ABA is ligated to PYR/PYL/RCAR proteins that interact with PP2Cs and inhibit their activity, in turn activating SnRK2, which phosphorylates ABF. *ABF* gene and protein expression is promoted by ABF themselves during ABA signaling. ABF promotes rapid BADH protein synthesis and triggers generation and accumulation of GB, which maintains osmotic balance to increase cassava tolerance to dehydration stresses.

To examine this possibility, in this study we assessed transcriptional expression of *MeBADH1* and *MeBADH2* in cassava (Fig. 7; Supplementary Tables S19 and S20). *MeBADH1* expression was induced in cassava leaves in response to drought and osmotic stresses, whereas *MeBADH2* levels were not affected. A similar pattern was observed in *Arabidopsis*, wherein one *BADH* family member was targeted to leucoplasts and involved in tolerance to high salt and drought, while another family member was targeted to peroxisomes and did not confer abiotic tolerance^72–74^. *MeBADH1* promoter sequences contained an ABRE, a cis-acting regulatory element that interacts with ABFs (Supplementary Table S18). Furthermore, in cassava leaves exposed to drought, osmotic, or salt stress, the *MeBADH1* expression pattern was concomitant with expression profiles of *MeABFs* (Figs 2 and 7; Supplementary Tables S5 and 21). Therefore, after various dehydration stress treatments, the expression of *MeABFs* in cassava plants may activate *MeBADH1* transcription by binding to the *MeBADH1* promoter in response to ABA signaling, and promote GB biosynthesis and accumulation by mediating increases in *MeBADH1* gene expression and MeBADH1 enzymatic activity. These responses could protect cassava plant cells from dehydration stresses by preserving the osmotic balance, thereby improving tolerance of cassava plants to dehydration stresses (Fig. 8).

## Conclusions

In summary, increasing evidence supports that ABF is an ABA-dependent transcription factor that regulates expression of downstream ABA-responsive genes in response to a variety of abiotic stresses in plants. In this study, we identified seven *MeABFs* that were distributed unevenly across six chromosomes, and clustered into three groups according to their phylogenetic relationships to *Arabidopsis* counterparts. Analysis of the 5′-upstream region of *MeABFs* revealed putative *cis*-acting elements related to hormone signaling, stress, and light responses, as well as circadian clock. Expression profiles of *MeABF* genes displayed clear differences among leaf, stem, root, and tuberous root tissues under normal and stress conditions, including drought, osmotic, or salt stress. Drought stress in cassava leaves and roots, osmotic stress in tuberous roots, and salt stress in stems induced expression of the highest number of *MeABFs* that showed significantly elevated expression. The GB content increased under drought, osmotic, or salt stress conditions in cassava leaves. BADH1 is involved in GB synthesis and the promoter sequence of *MeBADH1* contains an ABRE *cis*-acting element that would interact with ABFs. Furthermore, *MeBADH1* expression profiles were consistent with those for *MeABFs* in cassava leaves after the three stress treatments. These findings suggest that MeABFs activate *MeBADH1* transcription by binding to the *MeBADH1* promoter to induce MeBADH1 production that in turn promotes GB biosynthesis that can preserve the osmotic balance of cassava cells to provide tolerance to dehydration stresses.

## Methods

### Plant materials

Samples of cassava cultivar SC5 were obtained from the Institute of Tropical Bioscience and Biotechnology, Chinese Academy of Tropical Agricultural Sciences, China. To investigate the *MeABF* and *MeBADH* expression patterns and GB content in plantlet tissues in response to various treatments, 3-month-old cassava plants grown in plastic flower pots were used. For drought stress treatment, irrigation was withheld until no leaves remained on the plants. Since the plants lived for 18 days after drought treatment, we collected materials at 0 d, 3 d, 6 d, 9 d, 12 d, 15 d and 18 d after drought treatment. For osmotic or salt stress treatments, samples were collected at 0, 1, 2, 4, 8, 12, and 24 h after treatment with 400 mM NaCl or 30% PEG-6000^8^. For the control, cassava plants were watered on intervals of every three days. Experiments were carried out on three independent biological replicates.

After the experimental treatments, cassava plants were removed carefully from the plastic pots to allow collection of leaf, stem, root, and tuberous root tissues. All samples were frozen by immersion in liquid nitrogen immediately after collection and stored at −80 °C until analyzed.

### Isolation and identification of *MeABFs* and *MeBADHs*

To isolate *MeABF* and *MeBADH* genes, the nucleotide and amino acid sequences of *AtABFs* and *Arabidopsis thaliana BADHs* (*AtBADHs*) were downloaded from the database of TAIR (http://www.arabidopsis.org). The nucleotide and amino acid sequences of *MeABF* and *MeBADH* family members were searched using BLAST server and *AtABF* and *AtBADH* sequences as the query at Phyzotome (http://phytozome.jgi.doe.gov/pz/portal.html) database and NCBI (http://www.ncbi.nlm.nih.gov/) database^8^.

The cDNA sequences of likely *MeABF* and *MeBADH* genes in the cassava cultivar SC5 were cloned, sequenced, and then aligned with their counterparts from the cassava cultivar AM560-2. If these gene sequences showed more than 95% homology to those of the AM560-2, then identification was considered positive. Identified *MeABF* and *MeBADH* genes were reannotated and named.

### *In silico* analysis of *MeABFs* and *MeBADHs*

Phylogenetic and molecular evolutionary analyses of amino acid sequences of ABFs from cassava and *Arabidopsis* were performed using the MEGA 6.0 software^75^ by the neighbor-joining method^76^ with 1,000 bootstrap replicates.

Chromosomal locations of *MeABFs* were obtained using the BLAST program in Phyzotome websit (http://phytozome.jgi.doe.gov/pz/portal.html) and MapInspect software (http://mapinspect.software.informer.com) was subsequently used to draw the location images of *MeABFs*^8^.

To investigate the *cis*-elements in the promoter sequences of *MeABFs* and *MeBADHs*, the 1.5 kb genomic DNA sequences located the upstream of initiation codon (ATG) representing the core promoter regions of these genes were retrieved from the database of Phyzotome (http://phytozome.jgi.doe.gov/pz/portal.html). These sequences were converted to FASTA formatted sequences, and saved to pure text files. PlantCare software (http://bioinformatics.psb.ugent.be/webtools/plantcare/html) was used to identify the putative *cis*-acting regulatory elements located within these sequnces. Firstly, click the ‘Search for CARE’ button, and a new window will appear. Then, click the ‘Select file’ button, and submit the FASTA formatted sequences. Simultaneously, fill in the email address and the sequence name or ID. Finally, click the ‘Search’ button, and the predicted results will be obtained.

### Expression analysis of *MeABFs* and *MeBADHs*

Total RNA was extracted from the various tissues of cassava plants using the cetyltrimethyl ammonium bromide method^8^. Contaminating DNA in total RNA samples was eliminated by treatment with DNase I (RNase-free; Thermo Scientific, USA). First-strand cDNA was synthesized from 1.0 µg of total RNA using the first-strand cDNA synthesis kit according to the manufacturer’s instructions (Thermo Scientific, USA).

Gene-specific primer pairs (Supplementary Table S22) for quantitative real-time PCR (qRT-PCR) were designed using Primer Express Software v3.0 (Thermo Scientific, USA). The PCR mix for qRT-PCR contained 1.0 µl of diluted cDNA, 10 µl of 2× SYBR Green PCR Master Mix (Thermo Scientific, USA), and 200 nM of each gene-specific primer in a final volume of 20 µl. To determine the expression profiles of *MeABFs* and *MeBADHs* in cassava from stress-treated and control plants, qRT-PCR was performed using a Stratagene Mx3005P thermal cycler (Agilent Technologies, USA). Three independent biological replicates were performed for each time point of three stress treatments, with three technical replicates per qRT-PCR. The cassava *Actin* gene (Supplementary Tables S1 and S22) was used as reference gene for the normalization of RNA steady-state level, and the 2^−ΔΔCt^ method was used to calculate the relative expression values for the target genes^8^.

The log_2_ relative expression values were then visualized as heatmaps using the Multiple Experiment Viewer (MeV) software^8^. The absolute value of log_2_ relative expression values > 1.0 and *P* -value < 0.05 (determined by two-tailed Student’s *t* -test) was used as the threshold to assess the significant change in gene expression.

### Quantitation analysis of GB

GB content was measured using the commercially available plant GB assay kit (Cat. No. ml036338; Shanghai Enzyme-linked Biotechnology Co., Ltd., China). The GB content of fresh leaves from stress-treated and control cassava plants was measured using an enzyme-linked immunosorbent assay according to the manufacturer’s instructions. Statistical analysis was carried out using Student’s *t* -test and assigned *P* -value < 0.05, which was considered statistically significant.

## Supplementary information

Supplementary Fig. S1

Dataset 1
