# Supplementary material for: Involvement of abscisic acid-responsive element-binding factors in cassava (Manihot esculenta) dehydration stress response
Source: Sci Rep. 2019 Sep 2;9:12661. doi: 10.1038/s41598-019-49083-3 (PMC6718394; doi:10.1038/s41598-019-49083-3)
Supplement: Supplementary file 1 — Supplementary Fig. S1 [file 41598_2019_49083_MOESM1_ESM.docx]

Involvement of abscisic acid-responsive element-binding factors in cassava (*Manihot esculenta*) dehydration stress response

Ren-Jun Feng^1†^, Meng-Yun Ren^2†^, Li-Fang Lu^3†^, Ming Peng^1^, Xiao Guan^5^, Deng-Bo Zhou^1^, Miao-Yi Zhang^1^, Deng-Feng Qi^1^, Kai Li^1^, Wen Tang^1^, Tian-Yan Yun^4^, Yu-Feng Chen^4^, Fei Wang^1^, Dun Zhang^4^, Qi Shen^4^, Ping Liang^3*^, Yin-Dong Zhang^4*^, Jiang-Hui Xie^1*^

1 Key Laboratory of Biology and Genetic Resources of Tropical Crops, Ministry of Agriculture, Institute of Tropical Bioscience and Biotechnology, Chinese Academy of Tropical Agricultural Sciences (CATAS), Haikou 571101, P.R. China, 2 Institute of Crops and Nuclear Technology Utilization, Zhejiang Academy of Agricultural Sciences, Hangzhou 310021, P.R. China, 3 Department of Medical Physiology, Hainan Medical University, Haikou 571199, P.R. China, 4 College of Agronomy, Hainan University, Haikou 570228, P.R. China, 5 Chinese Research Academy of Environmental Sciences, Beijing 100012, P.R. China

^†^These authors contributed equally to this work.

* Correspondence: 804992279@qq.com (P.L.); 23300558@163.com (Y.D.Z.); 2453880045@qq.com (J.H.X.)

Supplementary Fig. S1

**Supplementary Fig. S1. Phylogenetic relationships of ABFs in cassava and *Arabidopsis*.** The neighbor-joining phylogenetic tree showing three distinct clusters as groups A, B, and C, was constructed using MEGA 6.0 program with 1,000 bootstrap replicates. Me, *Manihot esculenta*; At, *Arabidopsis thaliana*.
